# Frequent Use of the IgA Isotype in Human B Cells Encoding Potent Norovirus-Specific Monoclonal Antibodies That Block HBGA Binding

**DOI:** 10.1371/journal.ppat.1005719

**Published:** 2016-06-29

**Authors:** Gopal Sapparapu, Rita Czakó, Gabriela Alvarado, Sreejesh Shanker, B. V. Venkataram Prasad, Robert L. Atmar, Mary K. Estes, James E. Crowe

**Affiliations:** 1 Department of Pediatrics, Vanderbilt Medical Center, Nashville, Tennessee, United States of America; 2 Vanderbilt Vaccine Center, Vanderbilt Medical Center, Nashville, Tennessee, United States of America; 3 Department of Biochemistry and Molecular Biology, Baylor College of Medicine, Houston, Texas, United States of America; 4 Department of Pathology, Microbiology and Immunology, Vanderbilt Medical Center, Nashville, Tennessee, United States of America; 5 Department of Molecular Virology and Microbiology, Baylor College of Medicine, Houston, Texas, United States of America; 6 Department of Medicine, Baylor College of Medicine, Houston, Texas, United States of America; University of Chicago, UNITED STATES

## Abstract

Noroviruses (NoV) are the most common cause of non-bacterial acute gastroenteritis and cause local outbreaks of illness, especially in confined situations. Despite being identified four decades ago, the correlates of protection against norovirus gastroenteritis are still being elucidated. Recent studies have shown an association of protection with NoV-specific serum histo-blood group antigen-blocking antibody and with serum IgA in patients vaccinated with NoV VLPs. Here, we describe the isolation and characterization of human monoclonal IgG and IgA antibodies against a GI.I NoV, Norwalk virus (NV). A higher proportion of the IgA antibodies blocked NV VLP binding to glycans than did IgG antibodies. We generated isotype-switched variants of IgG and IgA antibodies to study the effects of the constant domain on blocking and binding activities. The IgA form of antibodies appears to be more potent than the IgG form in blocking norovirus binding to histo-blood group antigens. These studies suggest a unique role for IgA antibodies in protection from NoV infections by blocking attachment to cell receptors.

## Author Summary

Human noroviruses (HuNoV) have become the major etiologic agent of epidemic and sporadic acute gastroenteritis. There are currently no licensed vaccines or drugs to prevent or treat NoV infection. HuNoV infects people of all ages and, even though infection is characteristically acute and self-limiting, infection can become life threatening in children, the elderly, and the immunocompromised. Human challenge studies have shown that HuNoV infection elicits a robust humoral immune response and that antibodies that block virus-like particles (VLPs) from binding to host attachment factors are a correlate of protection. Using high-efficiency hybridoma technology, we isolated binding and blocking human monoclonal IgA antibodies (mAbs) and additional human IgG mAbs specific to GI.1 NoV. When comparing blocking efficiencies between both isotypes, we also found that human IgAs blocked GI.1 NoV VLPs from binding to synthetic host attachment factors more effectively than did IgGs. To design a vaccine that elicits broad protective immunity, we must have a solid understanding of the NoV-mediated human antibody response to infection. This study provides valuable insight into the human humoral response to NoV infection.

## Introduction

Norwalk virus, the prototype of human noroviruses (NoVs), was the first virus identified in 1972 as a causative agent for acute gastroenteritis [1]. NoVs are the leading cause of epidemic acute and sporadic cases of gastroenteritis responsible for about 19–21 million cases of infection leading to >70,000 hospitalizations and about 800 deaths annually in the U.S. [2]. NoVs recently surpassed rotaviruses as the leading cause of pediatric non-bacterial gastroenteritis after the introduction of vaccines against rotaviruses [3]. The infection is typically self-limiting, lasts for 1–3 days, and is characterized by diarrhea, vomiting, nausea, stomach pain and fever, with more severe complications and chronic disease in the immunocompromised. Therapy involves rest and rehydration, and no specific therapeutic agent is currently available.

NoVs, members of the *Caliciviridae* family, are non-enveloped and contain a positive-sense, non-segmented single stranded RNA genome enclosed by a protein capsid. The genome codes for three open reading frames (ORF), with the first ORF coding for six non-structural proteins involved in viral transcription and replication. The second and third ORFs encode virus protein 1 (VP1) and VP2, respectively. VP1 is a major capsid ~60 kDa protein and can self-assemble into virus-like particles (VLP) that resemble native virions both morphologically and antigenically [4]. The viruses are classified into at least six genogroups (GI, GII, GIII, GIV, GV and GVI), based upon the sequence of VP1 [5]. The genogroups are further subdivided into genotypes, with GI and GII accounting for the most diversity with 9 and 22 genotypes, respectively. GI and GII NoVs are responsible for the majority of human infections, with the genotype GII.4 responsible for most. Human susceptibility to NoVs depends on the expression of histo-blood group antigens (HBGAs) on the intestinal epithelial cells [6–8]. These blood group carbohydrates are thought to play a role as receptors or co-receptors based on recent studies of correlations between susceptibility, HBGA profile and secretor status (expression of secretor enzyme α1,2 fucosyltransferase) [2,9,10].

The correlates of protection in NoV infections are not completely understood. Almost all infected persons seroconvert, but epidemiological observations and clinical studies suggest that serum antibody measurable by traditional ELISA assays may not be long-lived or is otherwise insufficient to protect individuals from re-infection. Instead, the presence of anti-NoV Abs that block binding of virus to HBGA *in vitro* can protect from NoV gastroenteritis in the context of experimental challenge, suggesting a potential correlate [11,12]. We showed previously that a serum HBGA blocking antibody titer >200 [13] or a serum hemagglutination inhibition titer of >40 [14] is associated with protection of susceptible individuals from an experimental challenge. Deeper understanding of the immune response to human norovirus infection is hampered by the lack of a robust *in vitro* culture model and immunological reagents.

## Materials and Methods

### Ethics statement

Persons previously challenged experimentally with norovirus Hu/NoV/GI.1/Norwalk/68/US (Norwalk virus [NV]) were recruited to donate peripheral blood mononuclear cell (PBMC) samples for study.The protocol was reviewed and approved by the Baylor College of Medicine Institutional Review Board, and informed consent was obtained from the participants.

### Donors

We obtained PBMCs that were isolated from heparinized blood by density gradient centrifugation using Ficoll-Histopaque from donors 1–2 years following oral challenge with a GI.1 NV inoculum [15]. The donors from whom the panel of antibodies were isolated had been challenged 26 or 12 months prior. Infection was demonstrated in the laboratory by detection of viral genome and antigen in fecal samples, by RT-qPCR and ELISA, respectively. In addition, the donors demonstrated a greater than four-fold rise in serum antibody levels by total antibody ELISA and by HBGA blocking activity.

### Generation of EBV-transformed lymphoblastoid cell lines (LCLs) secreting NV-specific human monoclonal antibodies (mAbs)

B cells were transformed by infection with Epstein Barr virus (obtained from supernatant of cultured B95.8 cotton top tamarin lymphoblastoid line) in the presence of 2.5 μg/mL TLR agonist CpG (phosphorothioate-modified oligodeoxynucleotide ZOEZOEZZZZZOEEZOEZZZT, Life Technologies), 10 μM Chk2 inhibitor [Chk2i] (Sigma), 10 μg/mL cyclosporine A (Sigma) and plated in 384-well culture plates. After 7 days of culture, cells from one 384-well culture plate were expanded into four 96-well culture plates containing CpG, Chk2i and irradiated heterologous human PBMCs to serve as feeder layers for the growth of lymphoblastoid cell line (LCL) clusters. After an additional 3 days of culture, the supernatants were screened for binding to NV GI.1 VLP or disruption of the NV VLP–glycan interaction (described below). Briefly, 5 μL of supernatant from each well of transformed B cell cultures (in a total assay volume of 50 μL) were added to the wells coated with 1 μg/mL NV VLP. The bound antibodies were detected using alkaline phosphatase conjugated goat anti-human Ig (γ and αchain specific) (Southern Biotech). In blocking assays, 50 μL of diluted supernatant as described above were mixed with NV VLP and the complexes were added to H3-PAA (Glycotech, Rockville, MD) immobilized on neutravidin-coated plates, as described below. Supernatants from LCL cultures (diluted 1:10 in assay buffer) that had been selected for rotavirus-reactive antibody were used as negative controls.

### Generation of hybridomas secreting NV-specific mAbs from LCLs

Cells from wells with desired activity were subjected to electrofusion with HMMA2.5 myeloma cells. The fused cells then were cultured in a selective medium containing 100 μM hypoxanthine, 0.4 μM aminopterin, 16 μM thymidine (HAT Media Supplement, Sigma HO262), and 7 μg/mL ouabain (Sigma O3125) and incubated for 14–18 days before screening hybridomas for antibody production by ELISA. Cells from the positive wells were cloned biologically by sorting single cells into 384-well plates using a FACSAria III fluorescence-activated cell sorter (Becton Dickinson), cultured for about 14 days and screened for antibody production.

### Sequence analysis of antibody variable region genes

Total RNA was extracted from hybridoma cells and used for amplification of genes coding for the variable domains of the antibody clones. First-strand cDNA synthesis and RT-PCR were done with gene-specific primers as previously described (S1 Table) using the OneStep RT-PCR kit (Qiagen), according to the manufacturer’s protocols. The thermal cycling parameters were as follows: 50°C for 30 min, 95°C for 15 min, 39 cycles of (94°C for 1 min, 55°C for 1 min and 72°C for 1 min) followed by a final extension step for 10 min at 72°C. PCR products were purified using Agencourt AMPure XP magnetic beads (Beckman Coulter) and sequenced directly using an ABI3700 automated DNA sequencer without cloning. Heavy chain or light chain antibody variable region sequences were analyzed using the IMGT/V-Quest program [16,17]. The analysis involved the identification of germline genes that were used for antibody production, location of complementary determining regions (CDRs) and framework regions (FRs) as well as the number and location of somatic mutations that occurred during affinity maturation.

### Molecular engineering of antibody variable gene domains

For expression of recombinant forms of the antibody clones, the nucleotide sequences of variable domains were optimized for mammalian expression and synthesized (Genscript). The heavy chain fragments were cloned as EcoRI/HindIII fragments into pML-huCG1 or pML-huCA1 vectors for expression of γ1 or α1 chains, respectively [18]. The light chains were cloned as BglII/NotI fragments into pML-huCk or pML-huCL vectors for κ or λ chains, respectively.

### Production and purification of antibodies from hybridomas or from transfected HEK293 cells

For expression of antibodies from hybridoma clones, cells were cultured in serum-free medium, Hybridoma SFM (Life Technologies), for 21 days. Recombinant antibodies were expressed transiently in Expi293 F cells (Life Technologies), according to the manufacturer’s recommendation. Equal amounts of heavy and light chain DNA were used for transfections to generate recombinant IgG or monomeric IgA antibodies. For recombinant dimeric IgA, plasmids encoding cDNAs for the heavy chain, light chain and J chain DNA were mixed at 1:1:2 ratio as described [19]. Transfection was done using ExpiFectamine 293 transfection reagent (Life Technologies) according to the manufacturer’s protocols. After 7 days of culture, the supernatants were clarified by centrifugation and filtered using 0.4-μm pore size filter devices. Antibodies were harvested from the supernatants by affinity chromatography on HiTrap KappaSelect or LambdaSelect columns (Life Technologies) as previously described [19]. Antibodies eluted from affinity columns were concentrated using Amicon centrifugal filters (Millipore). Purified antibodies were resolved on polyacrylamide gels under reducing or non-reducing denaturing conditions and stained with Coomassie Blue reagent.

### Antibodies used as control reagents

We obtained polyclonal rabbit serum raised against NoV VLPs as a positive control for detection of VLPs coated on ELISA plates. This immune sera were generated by hyperimmunization of rabbits with NV VLPs as previously described [20]. We also prepared purified immunoglobulin from murine hybridoma cells secreting the mAbs 8812 or 3901. MAbs 8812 and 3901 were included in some receptor experiments as positive and negative controls for inhibition of NV VLP binding to receptor, based on previously determined activities [8]. We also used these murine mAbs as controls for immunoblotting experiments to determine if mAbs bound to linear epitopes, since mAb 3901, but not mAb 8812, has been described previously to bind linear epitopes [21]. In immunoblots, mAb 3901 binds denatured VP1 but mAb 8812 does not.

### Production and purification of VLPs

VLPs representing different norovirus genogroups (GI and GII) and genotypes (GI.1, NC_001959; GI.2, FJ515294; GI.4, GQ413970; GI.6, KC998959; GI.7, JN005886; GI.8, GU299761; GII.4; EU310927) were generated and purified as previously described [22]. Briefly, capsid proteins (VP1 and VP2) were expressed in SF9 insect cells (2.75x10^6 cells/mL of Grace’s insect cell media) from recombinant baculovirus expression vectors, and NoV VLPs were purified from culture supernatants on a cesium chloride gradient [20]. Structural integrity and purity of the VLP preparations were confirmed by electron microscopy of negatively stained VLPs (1.0% ammonium molybdenate (Sigma-Aldrich; St. Louis, MO), pH 6.0) on carbon coated grids and by Western blot, respectively. We also generated a GI.1 VLP (designated CT303) in which the P domain was deleted by mutagenesis of the VP1 gene construct [23]. We prepared a second mutated GI.1 VLP with the point mutation W375A that we previously determined ablates HBGA binding [24].

### VLP binding assay

Binding characterization of purified antibodies to NoV VLPs was carried out by ELISA. NoV VLPs were suspended in PBS at 1 μg/mL and coated in microwell plates (Nunc) for 16 h at 4°C, and the wells were blocked with 5% skim milk and 2% goat serum in PBS-Tween. Purified antibodies were diluted serially in PBS and added to the ELISA plates. The bound antibodies were detected using alkaline phosphatase conjugated goat anti-human κ or λ chain antibodies (Southern Biotech). To compare binding between different classes of antibodies, the concentrations of antibodies were adjusted to normalize for the binding sites (Fab = 1; IgG = 2; mIgA = 2 or dIgA = 4) before use in ELISA. The genotype specificity of antibody binding was determined by direct ELISA, as described above, with the following modifications: VLPs were coated at 10 μg/mL and antibodies were used at a concentration of 20 μg/mL. Plates were developed using ultra-TMB reagent (Pierce ThermoFisher; Rockford, IL), following the manufacturer’s protocol, and optical density as read at 450 nm using a SpectraMax M5 plate reader.

### P domain dimer specific binding assay

We prepared purified recombinant P domain dimeric protein, as previously described [24]. Briefly, a NV P domain construct was expressed in *E*. *coli* (Novagen) and purified by affinity chromatography, followed by size exclusion chromatography. We tested binding of each of the mAbs to P domain dimer by direct antigen ELISA, using the same protocol as described above for the VLP binding assay.

### Western blot

The nature of the epitopes bound by the human mAbs was determined by SDS-PAGE analysis and Western blot. NV VLPs were diluted in 5X Laemmli sample buffer and prepared for SDS-PAGE in one of the two following ways. Samples were either boiled at 100°C for 10 minutes or incubated at room temperature for 10 minutes prior to loading on separate pre-cast 14–20% polyacrylamide gels (Criterion TGX gel, BioRad; Hercules, CA) for electrophoresis. Electrophoresed proteins were transferred to nitrocellulose membrane for Western blot analysis. Human mAbs were diluted to 1 μg/mL in blocking solution (1% wt:vol, Kroger non-fat dried milk in 1X phosphate buffered saline). Two NV-reactive murine monoclonal antibodies (mAb 3901 and mAb 8812) and a Norwalk-reactive rabbit polyclonal were used as positive controls for detection of VP1. Blots were incubated overnight at 4°C. Bound antibodies were detected using either an anti-human Ig (A, G, M)-HRP, anti-mouse-HRP, or anti-rabbit-HRP conjugate antibody (Southern Biotech; Birmingham, AL). Blots were developed by chemiluminescence using West Pico HRP substrate (Pierce ThermoFisher; Rockford, IL) following the manufacturer’s instructions.

### HBGA blocking assay

Disruption of interaction between VLP and HBGAs was used as a surrogate assay for measuring NoV neutralization by human monoclonal antibodies. Pre-existing titer of HBGA blocking antibodies is correlated with protection from NoV gastroenteritis [11,13]. An HBGA blocking assay was carried out as previously described [11]. Briefly, biotin-polyacryamide (PAA)-blood group antigen conjugates (Glycotech, Rockville, MD) were immobilized on neutravidin-coated plates (Thermo Scientific). VLPs were mixed with serial dilutions of antibodies, and the complexes were added to the glycan-coated microtiter plates. The relative amount of VLP bound to HBGAs was determined using rabbit anti-NoV antiserum followed by horseradish peroxidase-conjugated goat anti-rabbit (Southern Biotech). We tested mAbs for inhibition of binding of NoV VLPs to additional biotin-PAA-HBGA ligands, including H type 1 (H1-PAA-biotin), H type 2 (H2-PAA-biotin), H type 3 (H3-PAA-biotin), A trisaccharide (tri-A-PAA-biotin), and Lewis(y) (Le(y)-PAA-biotin) (Glycotech, Rockville, MD). Plates were developed using ultra-TMB reagent (Pierce ThermoFisher; Rockford, IL), following the manufacturer’s protocol, and optical density as read at 450 nm using a SpectraMax M5 plate reader.

### Hemagglutination inhibition assay

Hemagglutination inhibition assays were performed as described previously [14]. In brief, Human type O erythrocytes were collected from a healthy adult in Alsever’s buffer, washed twice in Dulbecco’s phosphate-buffered saline (PBS) without Ca^2+^ or Mg^2+^, and pelleted by centrifugation at 500x*g* for 10 min at 4°C. Monoclonal antibodies (mAb; starting concentration 60 μg/mL for human mAb and 8.5 μg/mL for murine 8812) were diluted initially 1:10 in PBS with 0.85% saline (pH 5.5), and then serially 2-fold diluted. Four hemagglutination units (~2 ng) of Norwalk virus VLPs were mixed with the diluted monoclonal antibodies and incubated at room temperature for 30 min. The VLP-mAb mixture was added to an equal volume of 0.5% washed type O erythrocytes in 0.85% saline (pH 6.2) and incubated for 2 h at 4°C. The HAI titer was determined by identifying the reciprocal of the highest dilution of mAb that inhibited hemagglutination by the VLPs.

### Competition-binding ELISA analysis

Competition-binding ELISAs were carried out to determine whether the hmAbs we generated bound distinct or shared epitopes in the NV capsid protein. Briefly, each mAb was used to coat a 96-well microtiter plate (Greiner Bio-One; Monroe, NC) at a concentration of 2 μg/mL in carbonate coating buffer at 4°C overnight. Norwalk VLPs (100 ng/mL) were incubated with serial dilutions of each hMAb, ranging from 6.25 μg/mL to 250 μg/mL in assay buffer [1% non-fat dried milk (NFDM) in 1X PBS, w/v], for 2 hours at 37°C. Each plate included an antigen-only control to which no mAb had been added. The assay plate was washed three times with PBS containing 0.05% Tween 20 (PBS-T) and blocked for 1 hour at 37°C with 5% NFDM in PBS. The pre-incubated VLP/mAb preparations were added to the mAb-coated microtiter plate and plates were incubated for 2 hours at 37°C. Bound VLPs were detected using a rabbit anti-NV polyclonal antibody (1/10,000 in assay buffer; 2 hours at 37°C) followed by a commercial goat anti-rabbit-HRP conjugate antibody (Southern Biotech; 1/7500 in assay buffer; 45 minutes at 37°C). Plates were developed using ultra-TMB reagent (Pierce ThermoFisher; Rockford, IL), following the manufacturer’s protocol, and optical density as read at 450 nm using a SpectraMax M5 plate reader. Readings from duplicate wells were averaged. The percent competition for each competitor hMAb was calculated relative to the antigen-only control. MAbs were judged to compete for binding to the same site if maximum binding of the competing mAb was reduced to <25% of its un-competed binding. A level of 25–50% of its un-competed binding was considered intermediate competition.

## Results

### Isolation of NV-specific human IgG or IgA mAbs

**Fig 1:**
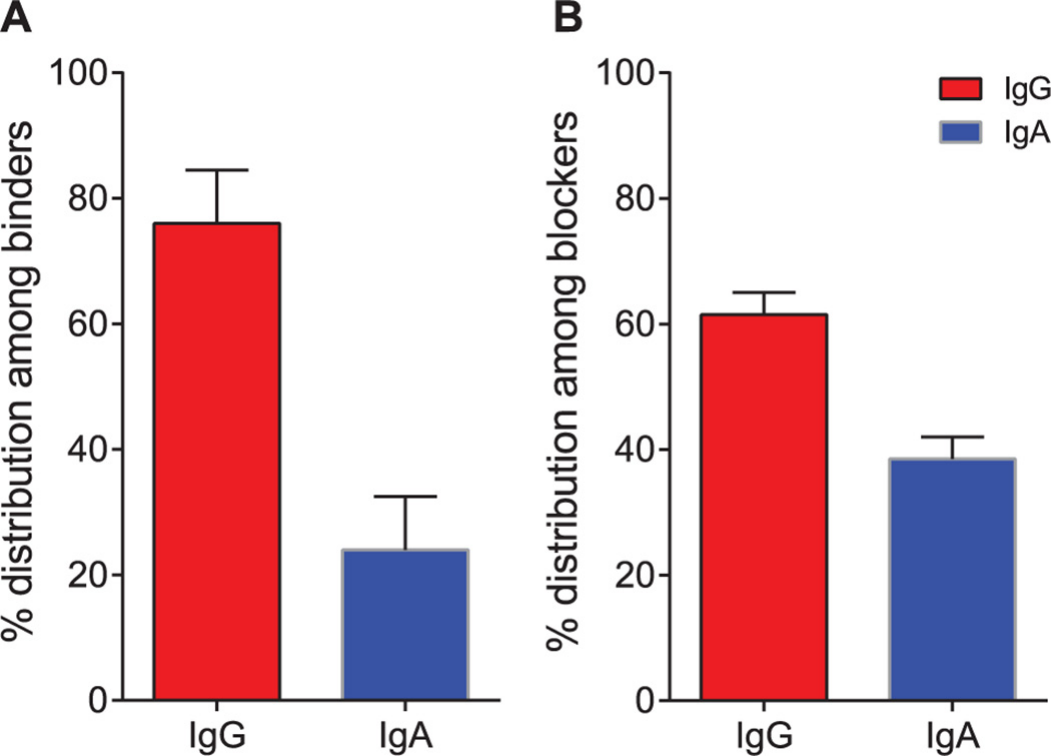
Screening supernatants of EBV-transformed B cell cultures from two NoV-challenged subjects. B cell culture supernatants were added to replicate microtiter plates coated with NoV VLP and probed with a mixture of (i) a mixture of anti-human (κ + λ; to determine the total number of binders), or (ii) anti-human IgG (γ-specific; to determine IgG frequency), or (iii) anti-human IgA (α-specific; to determine the IgA frequency) secondary antibodies. Blocking assay was done as described in Methods. The number of binding (A450 >1.5) and blocking (A450 <2.1) were counted and percent distribution among binders and blockers was calculated. Distribution of IgG (red) or IgA (blue) classes of antibodies that bound to NoV VLP (A) or blocked VLP—glycan interaction (B) is shown.

Currently there is not a robust method for growing NoVs, but studies suggest that blocking the interaction of VLP with glycan moieties can be used as a surrogate for neutralization activity [11,25]. We sought to isolate blocking mAbs to the NoV capsid protein from volunteers challenged with NV. PBMCs isolated from two NV-immune donors were transformed with EBV, and the LCL supernatants were screened for binding to NV VLPs and separately for blocking of binding to H3-PAA glycan. The transformed B cells from cell line supernatants exhibiting IgG or IgA binding to VLPs or blocking of VLPs to the glycan, or exhibiting both activities, were expanded. The supernatants from expanded LCLs then were assayed again for binding to VLPs, and bound antibodies were detected using either polyclonal anti-IgG (γ-specific) or anti-IgA (α-specific) secondary antibodies to determine the isotype of the binding antibodies. We used polyclonal secondary antibodies, instead of monoclonal antibodies, to minimize any differences in sensitivity of the secondary antibody to gamma or alpha chains and confirmed that the affinities of secondary antibodies did not differ measurably (S1 Fig). About 100 wells of the 384 wells tested were positive for NV binding. Of all the binding antibodies, a higher proportion of lines contained NV-specific antibodies that were IgG than IgA. However, the proportion of NV-binding antibodies that also exhibited blocking activity was higher for IgA antibodies than for IgG, suggesting that mAbs of the IgA isotype are highly over-represented in the repertoire of antibodies that block receptor binding (Fig 1). The cells in the positive wells were fused with a myeloma partner to generate a hybridoma clone. We were able to obtain a panel of seven IgG (1A8, 2L8, 3I23, 4E7, 4I23 from Donor 1 and NV1, NV48 from Donor 2) and seven IgA (2J3, 3I3, 4B19, 4C10, 5I2 from Donor 1 and NV41, NV56 from Donor 2) clones. The proper molecular assembly of IgG and dimeric IgA was confirmed by resolving antibodies on SDS-PAGE gels and staining with Coomassie Blue reagent (S2 Fig).

### IgA antibodies are more potent than IgG for receptor blocking

**Fig 2:**
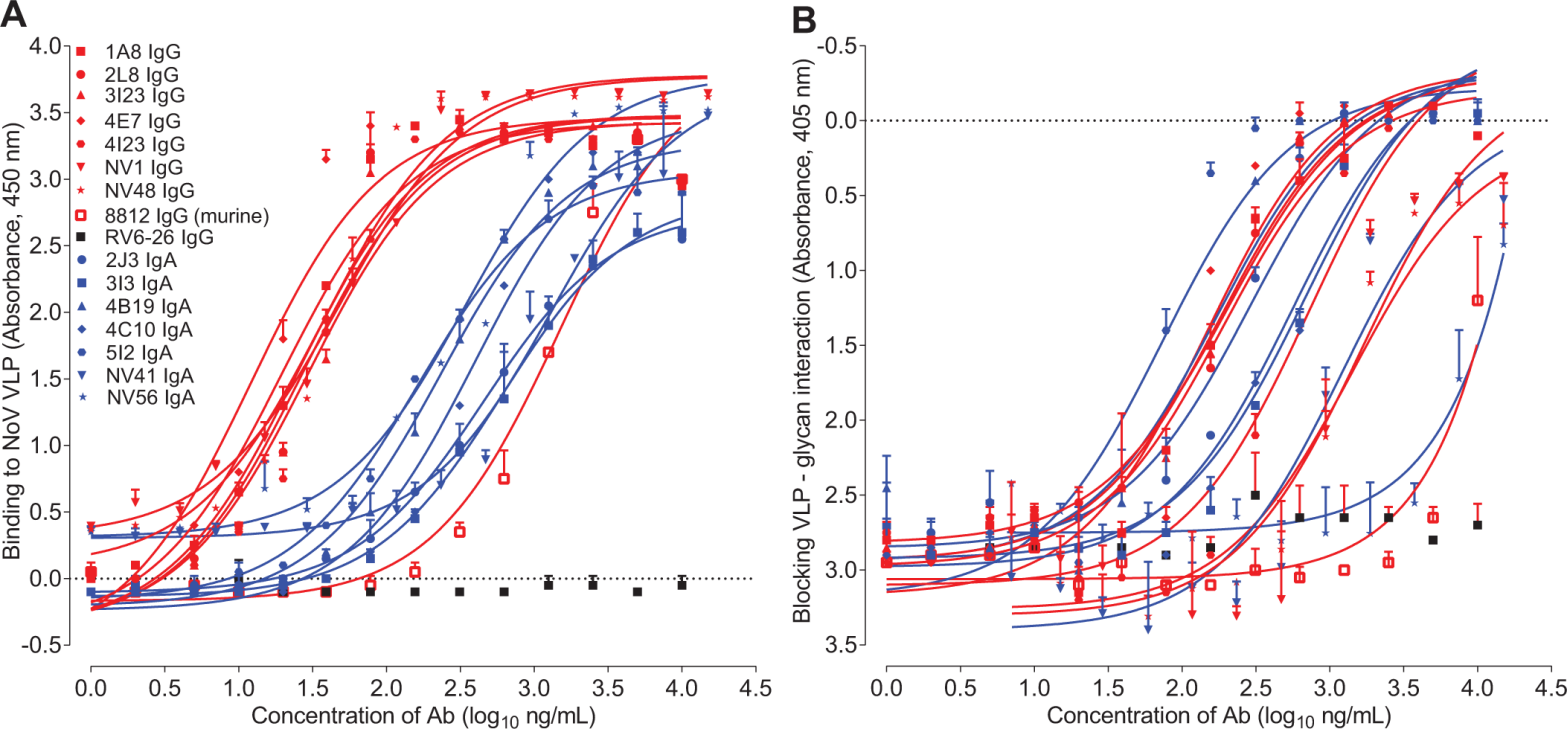
Binding and blocking characteristics of purified monoclonal antibodies. Purified IgG (red) or IgA (blue) antibodies were tested for binding to NV VLP in ELISA (A) or for blocking VLP—glycan interaction (B). Each of the IgG antibodies bound to VLPs with lower EC_50_ values than IgA antibodies, while in contrast the concentrations needed for blocking were similar for IgG and IgA. The blocking of murine mAb 8812 is shown in black.

Interpretation of the curves for Ig binding to VLPs was conducted after normalizing for the differing molarity of binding sites of IgG and dimeric IgA. IgA antibodies as a class appeared to have a lower affinity for binding in the VLP binding assays when compared with IgG. Interestingly, however, this class distinction was not apparent in the assays to detect antibody mediated blocking of VLP binding to glycan. These data suggest that even lower affinity IgA antibodies can mediate potent blocking activity (Fig 2). We constructed average binding and blocking curves for IgG and IgA sets of antibodies using R software package and generated representative binding and blocking curves for IgG and IgA (S3 Fig). The difference between binding of average IgG and average IgA was significant (p < 0.001), while the blocking was not significant (p = 0.39). We more fully characterized the antibodies obtained from Donor 1 in the experiments that follow.

### Genotype-specific recognition of NoV VLPs by mAbs

**Fig 3:**
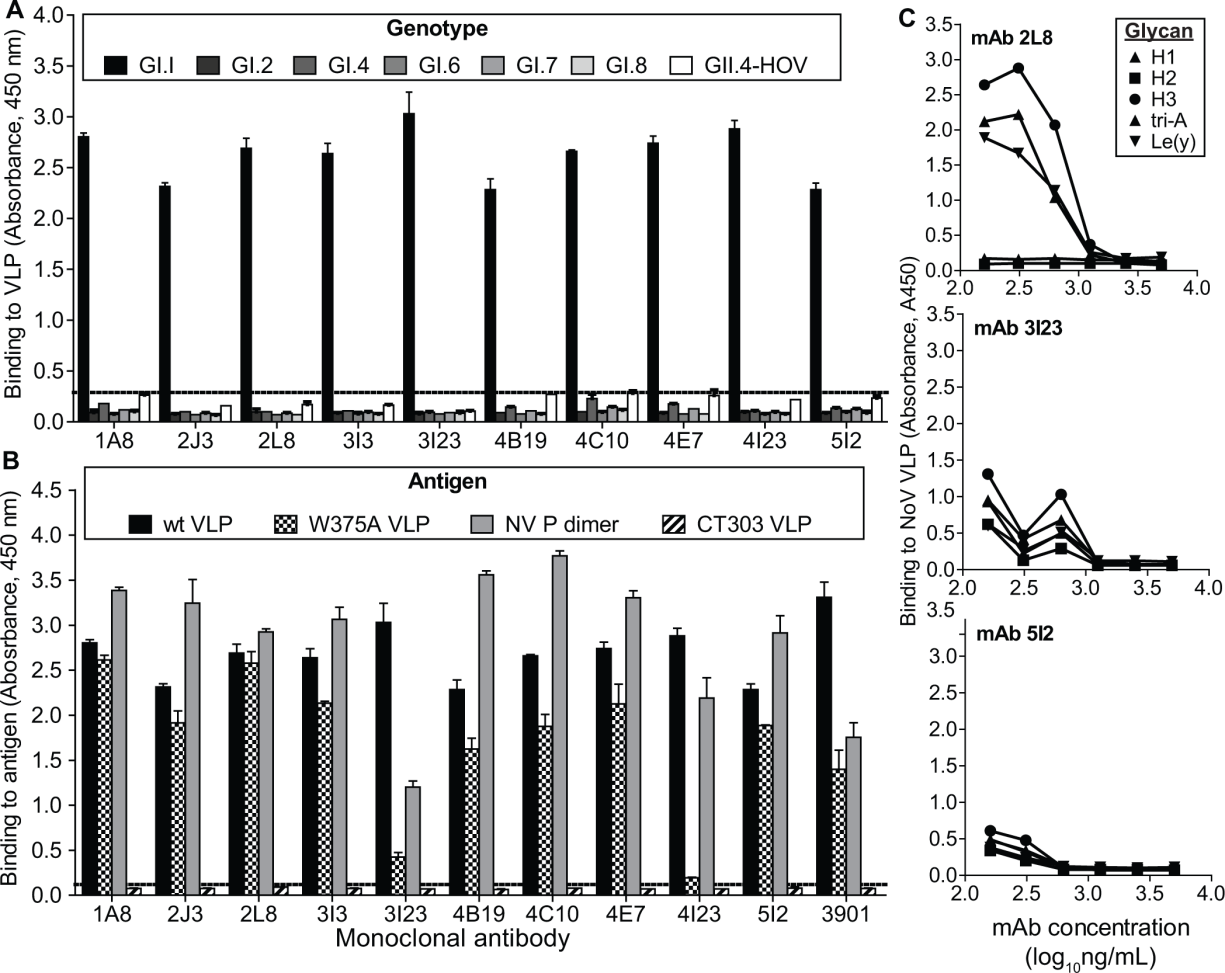
Specificity of human mAbs. The binding (mean absorbance at 450 nm ± SD) of purified mAbs at 20 μg/mL to VLPs representing homologous virus (NoV GI.I) or heterologous human NoVs of different genotypes (A) or antigens representing wild-type or mutant recombinant capsid proteins of homologous virus (B) were assessed by ELISA to evaluate genotype specificity and to infer the subdomain of major capsid protein bound by anti-norovirus mAbs. The data shown in each figure summarizes the results from 2 independent experiments. (C) The ligand specificity of mAb-mediated inhibition of NoV VLP binding to a panel of its glycan ligands (H1, H2, H3, tri-A or Le(y)) was evaluated in HBGA blocking assay for three mAbs, 2L8, 3I23 and 5I2.

The genotype specificity of mAb binding was assessed by direct antigen ELISA. VLPs representing different human NoV genotypes were coated on an ELISA plate. Each of the mAbs isolated bound Norwalk VLPs (GI.1) and none of the mAbs detected the other GI or GII NoV genotypes tested (Fig 3, panel A).

### MAbs bind to the P domain of the major capsid protein VP1

Direct antigen ELISA was performed for domain mapping of the human mAbs. All 10 hmAbs bound to wild-type NV VLPs, mediated by binding to the major capsid protein VP1 (Fig 3, panel B). The VP1 protein has two major domains, the highly conserved shell domain and the highly variable protruding (P) domain. Each of the 10 mAbs bound to recombinant P domain preparations, suggesting that their binding epitopes are contained within the P domain. Further support for this conclusion was provided by loss of mAb binding to VLPs assembled from a mutated VP1 (designated CT303) in which the P domain had been deleted [23,26]. We also tested NV VLPs with ablated HBGA binding through introduction of a point mutation (W375A) in the HBGA binding domain. Two of the mAbs failed to bind W375A VLPs, suggesting that the residue at this position influences VP1 recognition by mAbs 3I23 IgG and 4I23 IgG (Fig 3, panel B). We tested the specificity of three representative antibodies (2L8 IgG, 3I23 IgG and 5I2 IgA) to block VLP binding to diverse HBGA ligands, including H types 1, 2, 3 and Le(y). In every case the mAbs exhibited a strong inhibitory effect, except for 2L8 IgG, which had reduced activity to block binding to H type 3 tri-A and Le(y) (Fig 3, panel C).

### MAbs from a NV-infected individual bind nonlinear epitopes

**Fig 4:**
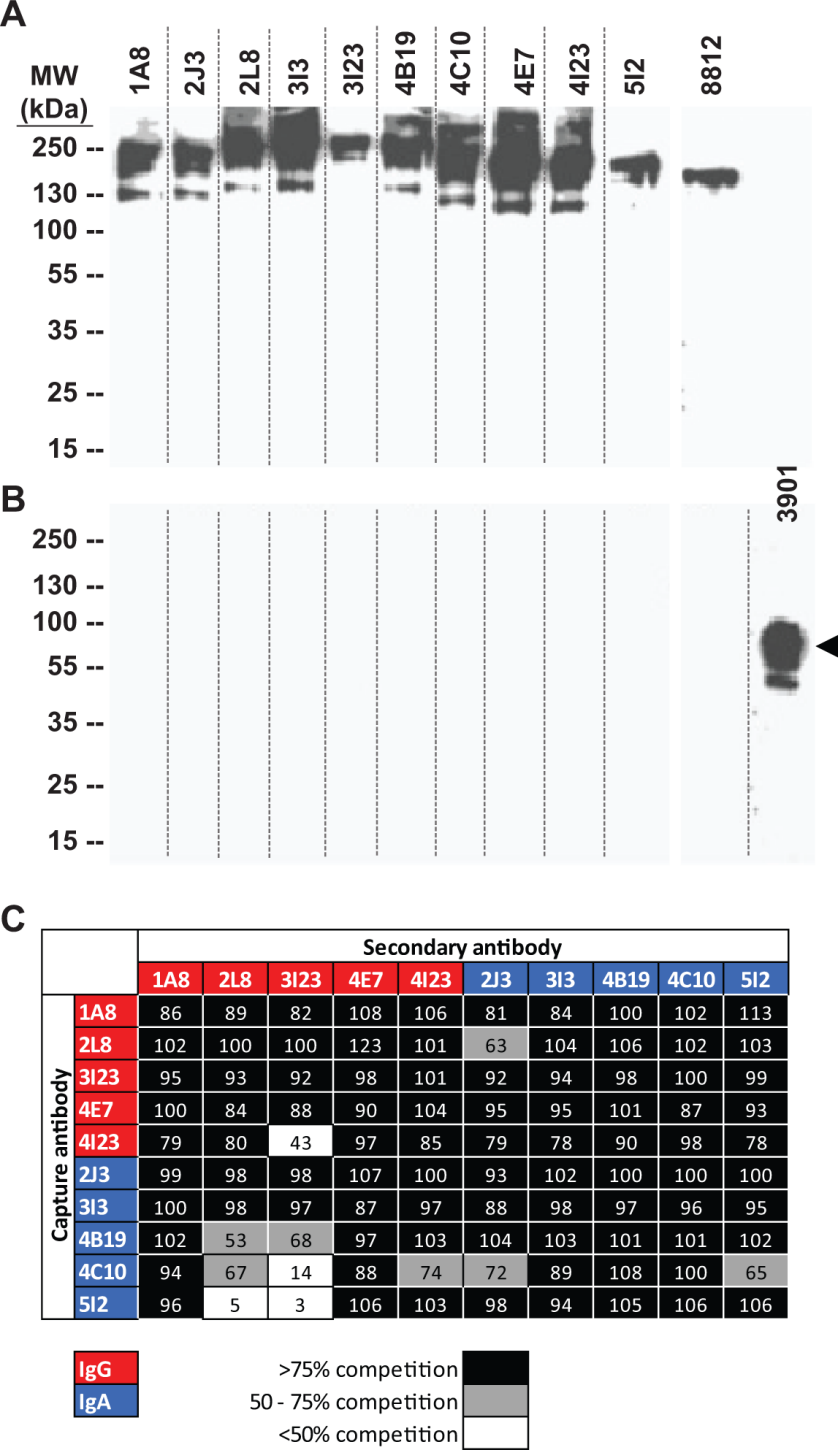
Nature of epitopes recognized by anti-norovirus mAbs. Norovirus VLPs were resolved on SDS-PAGE gels under (A) nonreducing, nondenaturing, or (B) reducing, denaturing conditions and the membranes were probed with anti-norovirus mAbs. All the human antibodies, and the murine mAb 8812, bound to conformational epitopes, while denatured VLP were bound only by mAb 3901. Arrowhead in panel B indicates VP1. (C) Antibodies were binned into competition-binding groups in ELISA as described in Methods. Most of the antibodies seem to compete for the same or spatially proximate epitopes. The asymmetric nature of competition suggests subtle factors such as the angle of approach of the antibodies seem to have an effect on competition.

To characterize the epitopes recognized by mAbs derived from Donor 1, we tested their ability to bind the NV major capsid protein VP1 by western blot (Fig 4, panels A and B). Murine mAbs 3901 or 8812 have been described previously to bind to linear or nonlinear epitopes, respectively, and were used as controls in this experiment [21]. Each of the 10 human mAbs and the murine mAb 8812 bound to unboiled preparations of NV VLPs, suggesting that the human mAbs recognize nonlinear epitopes in the major capsid protein VP1 (panel A). None of the human mAbs bound to denatured VP1 (panel B). Consistent with this finding, murine mAb 3901, but not murine mAb 8812, bound to denatured VP1.

### MAbs recognize at least 3 overlapping epitopes in VP1

Epitope binning was carried out by competition-binding ELISA. MAbs were assessed in a pairwise manner for their ability to inhibit binding of each other to NoV VLPs by ELISA (Fig 4, panel C). The observed patterns of competition-binding suggest that most of the mAbs bind to one major antigenic site. However, a few mAbs (2L8 IgG and 3I23 IgG) failed to inhibit capture of NV VLPs by other mAbs (4B19 IgA, 4C10 IgA, 4I23 IgG, 5I2 IgA). These observations suggest that members of the panel of mAbs bind to at least three distinct, but likely overlapping, epitopes on VP1.

### Isotype-switch variant IgG or IgA mAbs

Although IgA mAbs as a group appeared more potent in receptor blocking, that comparison is complicated by the fact that the IgA or IgG antibodies compared above with respect to blocking activity do not share the same variable regions. To determine if the variable domains of these antibodies contribute to the differences in activity, we analyzed the variable heavy and light chain genes of the antibodies, but did not find any unusual features in any of the antibody class in terms of gene families, mutation rate or CDR3 lengths (S2 Table). To study the effects of antibody isotype on functional activity in a more defined manner, we prepared IgG or IgA versions of representative blocking antibodies using mammalian cell recombinant expression of isotype-switch variant Ig molecules. We synthesized cDNAs coding for the variable domains after optimizing the sequence of the genes computationally for expression in mammalian cells. The heavy chain antibody variable genes were cloned in expression vectors for expression as γ or α chain. The light chain antibody variable genes were cloned in expression vectors for expression as κ or λ chains. Recombinant polymeric IgA was obtained by co-expression of joining (J) chain along with the heavy and light chains. Electrophoresis of purified proteins on SDS-PAGE gels under non-reducing conditions confirmed the correct assembly of IgG and dimeric IgA (S4 Fig). After normalizing for molarity of binding sites (IgG = 2, mIgA = 2, and dIgA = 4), each set of antibodies was tested in the binding and blocking assays. We calculated half-maximal effective concentrations (EC_50_) at which binding or blocking occurred. To enable comparison between the molecular forms of each antibody, we calculated the ratio of *blocking* EC_50_ to *binding* EC_50_ for each antibody. A lower ratio of blocking to binding indicated that smaller amounts of antibodies bound to VLP were needed for blocking the VLP binding to their receptor. In the antibodies we tested, mIgA and dIgA exhibited lower blocking-to-binding ratios than IgG (Table 1 and S5 Fig). Interestingly, the ratio was lower for mIgA than for IgG, despite these Igs having a similar molecular weight (150 kDa vs. 170 kDa). The activity of IgG versus IgA forms of the antibodies showed a similar trend in hemagglutination inhibition assays (S3 Table). This observation suggested that the higher potency of the IgA class of antibodies for blocking stems not only from their potential to make large polymeric Ig molecules with large capacity for steric hindrance following binding, but also from structural or functional features that are found even in the monomeric form of Ig molecules of that isotype.

**Table 1:** Blocking potencies of various isotypes of anti-NV mAbs.

Parent hybridoma	Recombinant isotype variants								
	EC_50_ (nM)			IC_50_ (nM)			IC_50_ / EC_50_ Ratio		
	IgG	mIgA	dIgA	IgG	mIgA	dIgA	IgG	mIgA	dIgA
**3I23 IgG**	2.7	2.3	1.6	19.7	9.8	4.9	7.3	4.3	3.1
**4I23 IgG**	0.6	2.2	2.1	19.6	11.5	17.5	32.7	5.2	8.3
**2J3 IgA**	0.4	0.6	0.6	17.9	15.3	11.9	44.8	25.5	19.8
**4C10 IgA**	1.4	2.1	1.4	13.9	9.2	9.6	9.9	4.4	6.9
**5I2 IgA**	3.3	3.2	2.4	32.1	18.2	12.4	9.7	5.7	5.2

IgG, monomeric IgA (mIgA) or dimeric IgA (dIgA) forms of anti-NoV mAbs were tested for binding to VLP and for blocking VLP-glycan interaction after adjusting for the number of valencies in each moiety (IgG and mIgA = 2; dIgA = 4). The concentration at which half-maximal binding (EC_50_) or inhibition (IC_50_) occurred was calculated from non-linear regression analysis. The ratio of IC_50_ to EC_50_ suggests that more IgG is needed for blocking activity compared to mIgA or dIgA for all the three antibodies compared.

## Discussion

The molecular basis of antibody-mediated inhibition of human NoV infection is poorly understood. It has been difficult to study NoV neutralization because of the lack of a robust cell culture system for growing virus. Recent studies suggest, however, that the presence of antibodies that block NoV-HBGA interactions is associated with protection against illness [13,20,24]. In this model, antibodies that disrupt the interaction of NoV VLPs with HBGA ligands thus act as putative virus neutralizing antibodies. The structural and functional features of these putative neutralizing antibodies are not known. Blocking activity of purified, serum-derived IgA antibodies was recently described, and our group recently identified serum IgA and salivary IgA antibodies as novel correlates of protection from NoV gastroenteritis [12,27,28]. In the studies presented here, we isolated a panel of hmAbs with potent NoV-HBGA blocking activity, representing IgA and IgG isotypes, from an immune individual following experimental virus challenge. The features of these antibodies reveal new aspects of antibody-mediated NoV inhibition.

The most interesting finding from these detailed studies is that naturally-occurring NoV-specific human antibodies of the IgA isotype exhibit enhanced potency for receptor blocking, compared to IgG antibodies isolated in a similar fashion. These data suggest that this enhanced potency stems from two principal factors. First, there appears to be some intrinsic structural or functional features of the IgA isotype that confer enhanced blocking activity, even in monomeric forms of IgA antibodies, compared to a matched IgG variant. It is known that IgG and IgA molecules differ in certain functional aspects, due to sequence polymorphisms in the constant domain[29,30]. In fact, previous studies with antibodies to other microbial agents have suggested that polymorphisms in the constant regions even of differing IgG subclasses can mediate a profound phenotypic change in the pattern binding of antibodies [31]. We did not determine the molecular basis for this effect against NV virus, but the enhancement is of interest as it has relevance to both antibody and vaccine design efforts.

Second, we found that dimeric IgAs exhibited enhanced potency for blocking compared to matched monomeric IgA or IgG counterparts. Most likely, this finding is due to the large molecular weight of dimeric IgA, which probably facilitates a more profound receptor blocking capacity.

It was interesting that many of the B cells we isolated from blood that encoded NoV-specific IgA secreted polymeric IgA in the naturally-occurring form. It has been noted previously that secreted IgA proteins in the serum typically are almost exclusively monomeric. However, we did not study serum antibodies here; rather we isolated NoV-specific IgA-encoding B cells from the blood, and many of these secreted dimeric IgA after recovery. It is possible that these cells are circulating in peripheral blood en route to mucosal tissues. The technique that we used predominantly isolates memory B cells, and we isolated these cells during the convalescent phase from the donor. Therefore, it is not anticipated that the cells would have been secreting dimeric IgA into the serum in the donor.

We performed sequence analysis of the antibody variable genes encoding these mAbs. The data show that there is a wide diversity of antibody variable genes that encode antibodies that block receptor binding. This finding is encouraging, because it suggests that there is no genetic restriction on the ability of diverse humans to make receptor-blocking antibodies to NoV. We examined the genetic features of the antibodies to see if there were any unusual characteristics. Some especially important domains in viral surface proteins that are susceptible to recognition by potent virus-neutralizing antibodies, such as HIV envelope or influenza hemagglutinin, are associated with unusual genetic and structural features such as long heavy chain CDR3 regions or a very high level of somatic mutations [32–34]. We found that the NoV receptor-blocking antibodies did not possess any extreme genetic features. Diverse antibody variable genes were used, and the level of somatic mutation observed was typical of that found in human memory B cells [35,36]. The length of heavy chain CDR3 regions was average, and there was no unusual occurrence of insertions or deletions.

Human NoVs cause acute gastroenteritis worldwide, and they exhibit a high degree of genetic variation in different geographical locations. Field strains of NoV evolve, and dominant novel strains emerge periodically that exhibit antigenic variation and differential glycan-binding specificities due to genetic changes that alter structure in the P domain of the NoV capsid protein [37,38]. HBGAs most likely function as co-receptors or cell attachment factors to NoVs, and thus they determine susceptibility to infection [10,37,39]. The structural basis for the interaction of the P domain of the NoV capsid protein with HBGAs is fairly well understood, but how particular sequence polymorphisms in this domain determine the effect on genogroup-specific biology is less well understood. We also do not understand how such sequence variations mediate binding or escape from human neutralizing antibodies. Further study of the mAbs that we isolated here should prove useful in this regard. These antibodies exhibited GI.1 NoV specificity, consistent with the background of the virus that was used for the donor challenge and with the antigen used to screen for the NoV-specific antibodies. Structural studies of the interaction of antigen-antibody complexes with these mAbs could elucidate the critical residues in the P domain that contribute to antibody recognition in a genogroup-specific manner. Further studies also would allow us to understand the mechanism by which NoV-neutralizing antibodies block HBGA binding, whether it is by directly binding or altering the conformation of the HBGA binding site or by sterically hindering access to the HBGA binding site [40]. Such studies will shed light on additional factors that regulate protection from human NoV infection and disease.

## Supporting Information

S1 Fig Detection of IgA or IgG antibodies with a common conjugated antibody. ELISA plates were coated with one of the ten human monoclonal antibodies in varying dilutions and then detected with the polyclonal goat antihuman secondary antibody suspension. IgA antibodies are shown in red, IgG antibodies in black.
(PDF)

S2 Fig Molecular assembly of hybridoma-derived antibodies obtained from Donor 1. IgA (blue) and IgG (red) antibodies were purified by affinity chromatography and resolved on SDS polyacrylamide gels under reducing, denaturing conditions (panel A) or non-reducing conditions (panel B) and stained with Coomassie Blue. Monomeric (*) and dimeric (**) forms of IgA are evident.
(PDF)

S3 Fig Average binding and blocking profiles of IgG and IgA antibodies. The dose-response curves for binding (A) or blocking (B) for all IgG and IgA antibodies were averaged to generate representative curves for each class using R software package. The binding curves for IgG and IgA are significantly different (p < 0.001), while there is insufficient evidence to show that IgG and IgA differ in blocking (p = 0.39).
(PDF)

S4 Fig Variable heavy and light chain domains of anti-NoV mAbs were cloned into expression vectors containing γ or α constant domains for heavy chains, and κ or λ constant domains for the light chains. Antibodies were expressed transiently in HEK293 cells. For expression of dIgA, a plasmid coding for J chain was cotransfected with the heavy and light chains. Antibodies purified from supernatant by affinity chromatography were resolved on SDS-PAGE gels under nonreducing conditions and stained with Coomassie Blue reagent.
(PDF)

S5 Fig Representative curves for blocking assays with isotype switch variants for each of the antibody clones. IgG or monomeric (mIgA) or dimeric (dIgA) forms of IgA were used in the HBGA blocking assay. Results are shown with concentration of Ab as log_10_ nM combining sites).
(PDF)

S1 Table Primers used in RT-PCR for amplifying heavy or light chain antibody variable genes. (PDF)

S2 Table Genetic characteristics of anti-norovirus mAbs. (PDF)

S3 Table Hemagglutination inhibition activity for recombinant isotype switch variants. Hemagglutination inhibition assays were performed as described previously [14]. In brief, human type O erythrocytes were collected from a healthy adult in Alsever’s buffer, washed twice in Dulbecco’s phosphate-buffered saline (PBS) without Ca2+ or Mg2+, and pelleted by centrifugation at 500 x *g* for 10 min at 4°C. Monoclonal antibodies (mAb; starting concentration 60 μg/mL) were diluted initially 1:10 in PBS with 0.85% saline (pH 5.5), and then serially 2-fold diluted. Four hemagglutination units (~2 ng) of Norwalk virus VLPs were mixed with the diluted monoclonal antibodies and incubated at room temperature for 30 min. The VLP-mAb mixture was added to an equal volume of 0.5% washed type O erythrocytes in 0.85% saline (pH 6.2) and incubated for 2 h at 4°C. The HAI titer was determined by identifying the reciprocal of the highest dilution of mAb that inhibited hemagglutination by the VLPs.
(PDF)
