# Supplementary material for: Frequent Use of the IgA Isotype in Human B Cells Encoding Potent Norovirus-Specific Monoclonal Antibodies That Block HBGA Binding
Source: PLoS Pathog. 2016 Jun 29;12(6):e1005719. doi: 10.1371/journal.ppat.1005719 (PMC4927092; doi:10.1371/journal.ppat.1005719)
Supplement: S1 Fig — ELISA plates were coated with one of the ten human monoclonal antibodies in varying dilutions and then detected with the polyclonal goat antihuman secondary antibody suspension. IgA antibodies are shown in red, IgG antibodies in black. (PDF) [file ppat.1005719.s001.pdf]

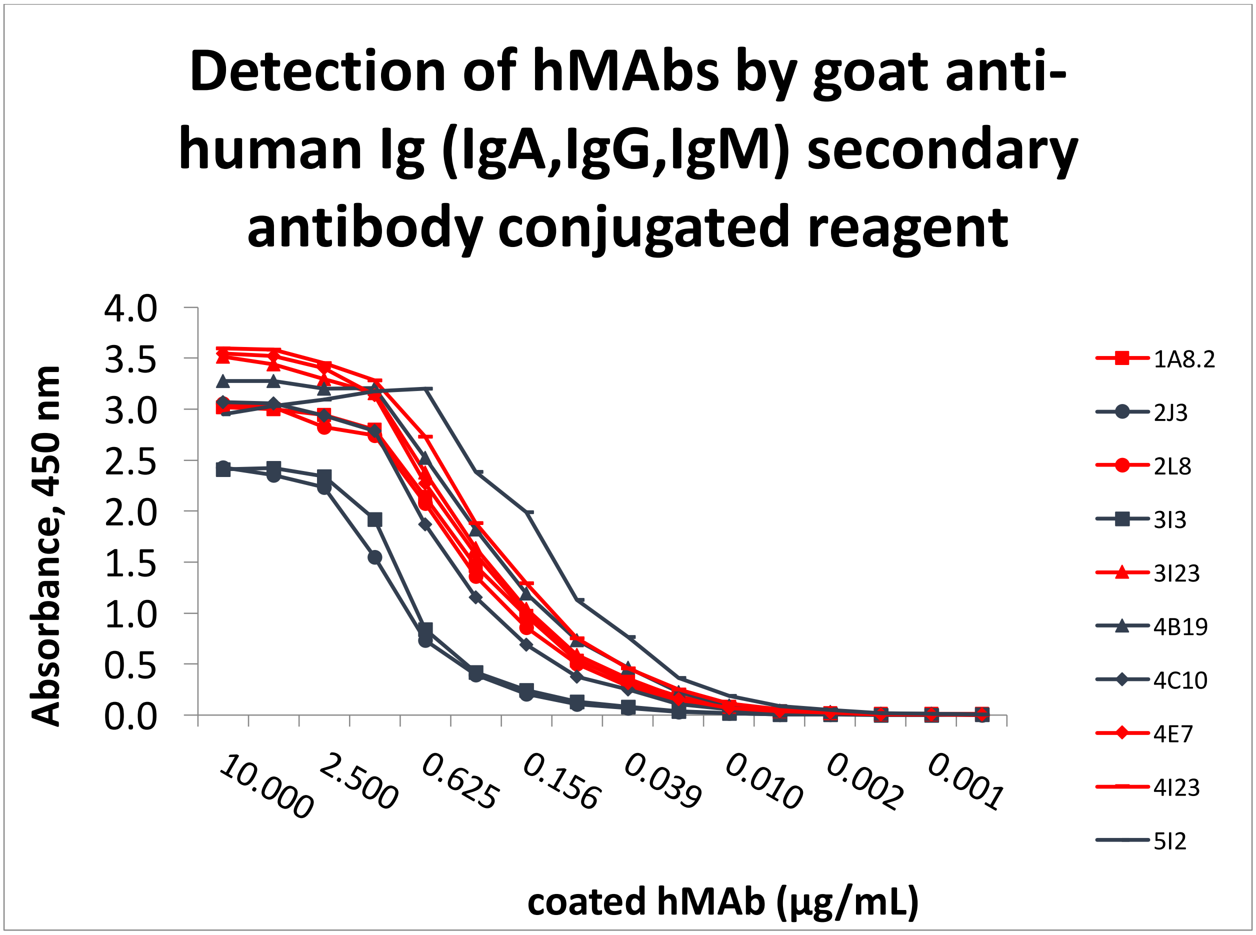

Figure S1. Detection of IgA or IgG antibodies with a common conjugated antibody. ELISA plates were coated with one of the ten human monoclonal antibodies in varying dilutions and then detected with the polyclonal goat anti-human secondary antibody suspension. IgA antibodies are shown in red, IgG antibodies in black.
