# Supplementary material for: Frequent Use of the IgA Isotype in Human B Cells Encoding Potent Norovirus-Specific Monoclonal Antibodies That Block HBGA Binding
Source: PLoS Pathog. 2016 Jun 29;12(6):e1005719. doi: 10.1371/journal.ppat.1005719 (PMC4927092; doi:10.1371/journal.ppat.1005719)
Supplement: S2 Fig — IgA (blue) and IgG (red) antibodies were purified by affinity chromatography and resolved on SDS polyacrylamide gels under reducing, denaturing conditions (panel A) or non-reducing conditions (panel B) and stained with Coomassie Blue. Monomeric (*) and dimeric (**) forms of IgA are evident. (PDF) [file ppat.1005719.s002.pdf]

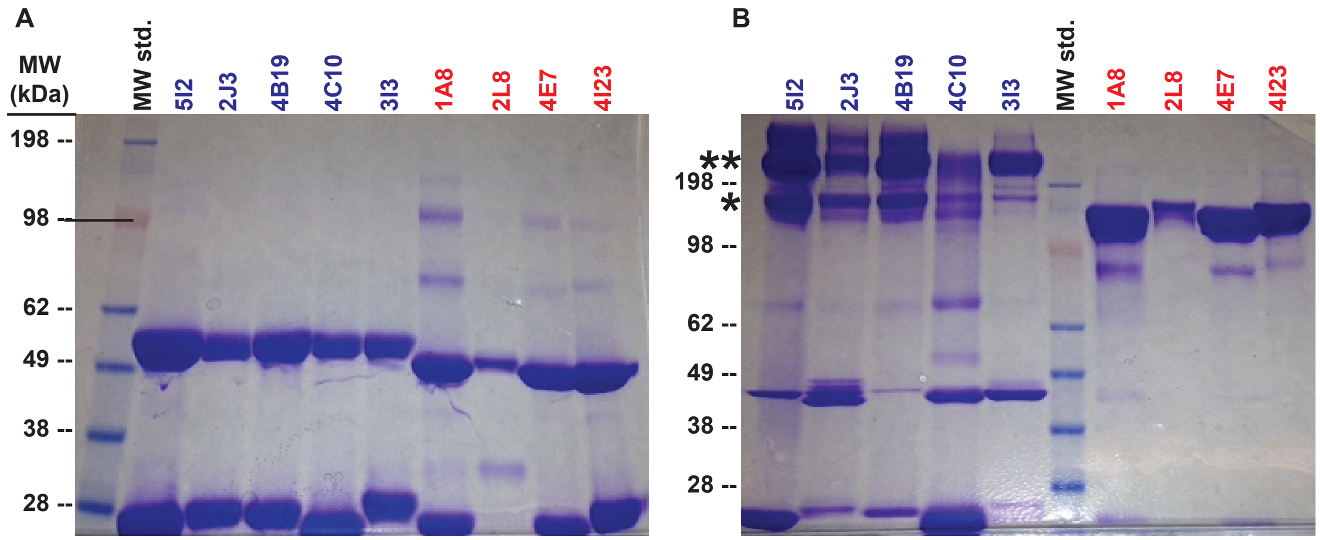

Figure S2. Molecular assembly of hybridoma-derived antibodies obtained from Donor 1. IgA (blue) and IgG (red) antibodies were purified by affinity chromatography and resolved on SDS polyacrylamide gels under reducing, denaturing conditions (panel A) or non-reducing conditions (panel B) and stained with Coomassie Blue. Monomeric (*) and dimeric (**) forms of IgA are evident.
