# Supplementary material for: Frequent Use of the IgA Isotype in Human B Cells Encoding Potent Norovirus-Specific Monoclonal Antibodies That Block HBGA Binding
Source: PLoS Pathog. 2016 Jun 29;12(6):e1005719. doi: 10.1371/journal.ppat.1005719 (PMC4927092; doi:10.1371/journal.ppat.1005719)
Supplement: S3 Fig — The dose-response curves for binding (A) or blocking (B) for all IgG and IgA antibodies were averaged to generate representative curves for each class using R software package. The binding curves for IgG and IgA are significantly different (p < 0.001), while there is insufficient evidence to show that IgG and IgA differ in blocking (p = 0.39). (PDF) [file ppat.1005719.s003.pdf]

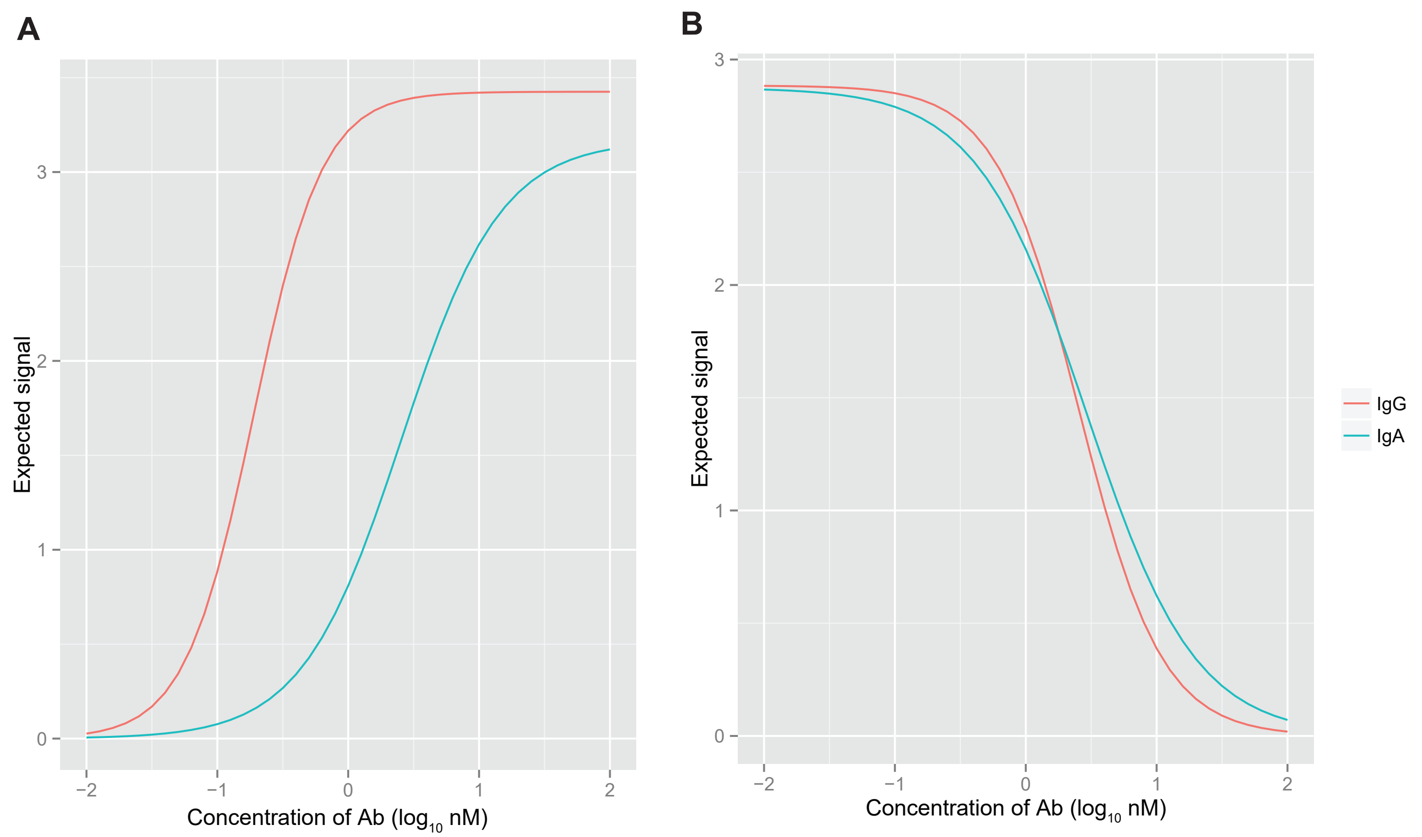


**Figure S3. Average binding and blocking profiles of IgG and IgA antibodies.** The dose-response curves for binding (A) or blocking (B) for all IgG and IgA antibodies were averaged to generate a representative curves for each class using R software package. The binding curves for IgG and IgA are significantly different ($p < 0.001$), while there is insufficient evidence to show that IgG and IgA differ in blocking ($p = 0.39$).
