# Supplementary material for: Frequent Use of the IgA Isotype in Human B Cells Encoding Potent Norovirus-Specific Monoclonal Antibodies That Block HBGA Binding
Source: PLoS Pathog. 2016 Jun 29;12(6):e1005719. doi: 10.1371/journal.ppat.1005719 (PMC4927092; doi:10.1371/journal.ppat.1005719)
Supplement: S4 Fig — Antibodies were expressed transiently in HEK293 cells. For expression of dIgA, a plasmid coding for J chain was cotransfected with the heavy and light chains. Antibodies purified from supernatant by affinity chromatography were resolved on SDS-PAGE gels under nonreducing conditions and stained with Coomassie Blue reagent. (PDF) [file ppat.1005719.s004.pdf]

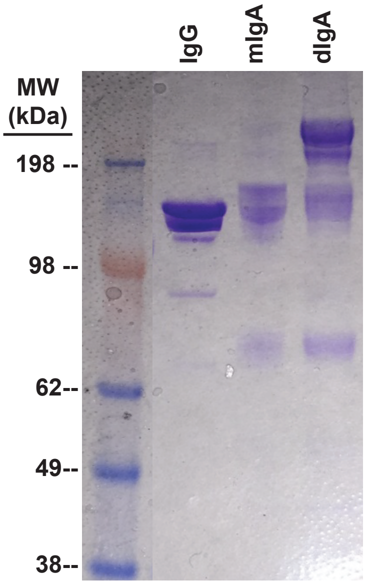

Figure S4. Variable heavy and light chain domains of anti-NoV mAbs were cloned into expression vectors containing gamma or alpha constant domains for heavy chains, and kappa or lambda constant domains for the light chains. Antibodies were expressed transiently in HEK293 cells. For expression of dIgA, a plasmid coding for J chain was cotransfected with the heavy and light chains. Antibodies purified from supernatant by affinity chromatography were resolved on SDS-PAGE gels under nonreducing conditions and stained with Coomassie Blue reagent.
