# Supplementary material for: Frequent Use of the IgA Isotype in Human B Cells Encoding Potent Norovirus-Specific Monoclonal Antibodies That Block HBGA Binding
Source: PLoS Pathog. 2016 Jun 29;12(6):e1005719. doi: 10.1371/journal.ppat.1005719 (PMC4927092; doi:10.1371/journal.ppat.1005719)

**Figure S5.** Representative curves for blocking assays with each of the antibody clones

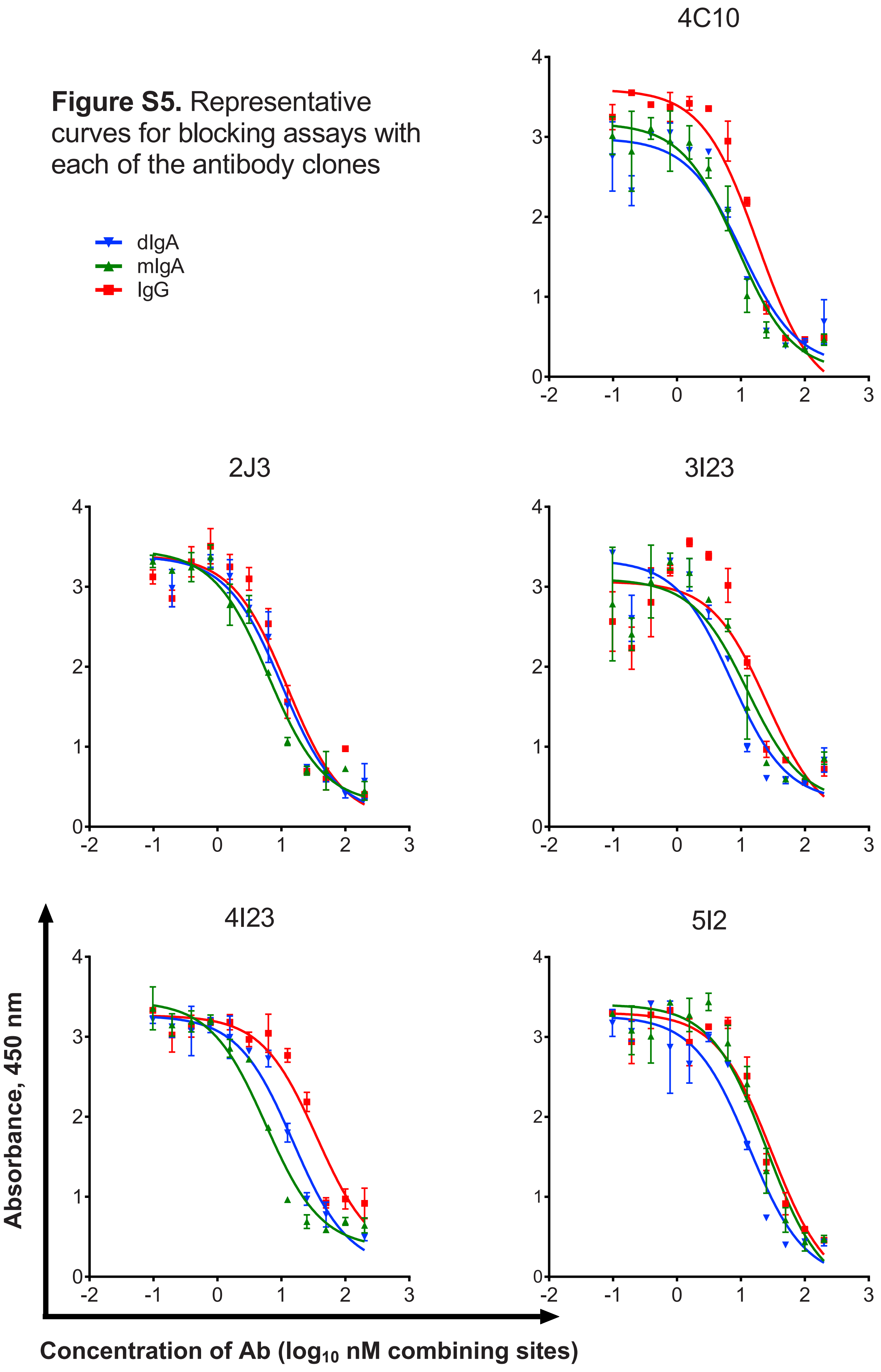

Supplement: S5 Fig — IgG or monomeric (mIgA) or dimeric (dIgA) forms of IgA were used in the HBGA blocking assay. Results are shown with concentration of Ab as log10 nM combining sites). (PDF) [file ppat.1005719.s005.pdf]
