# Supplementary material for: Frequent Use of the IgA Isotype in Human B Cells Encoding Potent Norovirus-Specific Monoclonal Antibodies That Block HBGA Binding
Source: PLoS Pathog. 2016 Jun 29;12(6):e1005719. doi: 10.1371/journal.ppat.1005719 (PMC4927092; doi:10.1371/journal.ppat.1005719)
Supplement: S1 Table — (PDF) [file ppat.1005719.s006.pdf]

Table S1. Primers used in RT-PCR for amplifying heavy or light chain antibody variable genes

Oligo Name	Sequence 5' to 3'
IgExp_H1	GTTTTAAAAGGTGTCCTGTGTCARRTNCAGCTGGTRCAGTC
IgExp_H2	GTTTTAAAAGGTGTCCTGTGTCAGRTCACCTTGARGGAGTC
IgExp_H3	GTTTTAAAAGGTGTCCTGTGTSARGTGCAGCTGGTGGAGTC
IgExp_H4	GTTTTAAAAGGTGTCCTGTGTCAGSTGCAGCTRSAGGAGTC
IgExp_H5	GTTTTAAAAGGTGTCCTGTGTGARGTGCAGCTGGTGCAGTC
IgExp_H6	GTTTTAAAAGGTGTCCTGTGTCAGGTACAGCTGCAGCAGTC
IgExp_H7	GTTTTAAAAGGTGTCCTGTGTCAGGTGCAGCTGGTGCAGTC
IgExp_Hconst	GATGGGCCCTTGAAGCTTGCTGAGGAGACGGTGACCAGGGT
IgExp_K1	GAATCCCAGGCATGAGATCTGMCATCCRGWTGACCCAG
IgExp_K2	GAATCCCAGGCATGAGATCTGAKRTTGTGATGACYCAG
IgExp_K3	GAATCCCAGGCATGAGATCTGAAATWGTRWTGACRCAG
IgExp_K4	GAATCCCAGGCATGAGATCTGACATCGTGATGACCCAG
IgExp_K5	GAATCCCAGGCATGAGATCTGAAACGACACTCACGCAG
IgExp_K6	GAATCCCAGGCATGAGATCTGAWRTTGTGMTGACWCAG
IgExp_Kconst	GATGGCGGGAAGATGAAGACAGATGGTGCGGCCGCAGT
IgExp_L1	GAATCCCAGGCATGAGATCTCAGTCTGTSBTGACKCAG
IgExp_L2	GAATCCCAGGCATGAGATCTCARTCTGCCCTGACTCAG
IgExp_L3	GAATCCCAGGCATGAGATCTTCCTMTGDGCYRAYWCAG
IgExp_L4	GAATCCCAGGCATGAGATCTCWGCYTGTGCTGACTCAA
IgExp_L5	GAATCCCAGGCATGAGATCTCAGSCTGTGCTGACTCAG
IgExp_L6	GAATCCCAGGCATGAGATCTAATTTTATGCTGACTCAG
IgExp_L7	GAATCCCAGGCATGAGATCTCAGRCTGTGGTGACTCAG
IgExp_L8	GAATCCCAGGCATGAGATCTCAGWCTGTGGTGACCCAG
IgExp_L9	GAATCCCAGGCATGAGATCTCAGCCTGTGCTGACTCAG
IgExp_L10	GAATCCCAGGCATGAGATCTCAGGCAGGGCTGACTCAG
IgExp_L11	GAATCCCAGGCATGAGATCTCGGCCCGTGCTGACTCAG
IgExp_Lconst	AGGGGGGAACAGAGTGACASTTGGAGCGGCCTTAGGCTG
