# Supplementary material for: Frequent Use of the IgA Isotype in Human B Cells Encoding Potent Norovirus-Specific Monoclonal Antibodies That Block HBGA Binding
Source: PLoS Pathog. 2016 Jun 29;12(6):e1005719. doi: 10.1371/journal.ppat.1005719 (PMC4927092; doi:10.1371/journal.ppat.1005719)
Supplement: S2 Table — (PDF) [file ppat.1005719.s007.pdf]

Table S2. Genetic characteristics of anti-norovirus mAbs

mAb	Class and isotype	EC_{50} (ng/mL)	Heavy chain									Light chain							
			V gene	J gene	D gene	Number of AA in			HCDR3 AA sequence	Nt mutations	AA mutations	V gene	J gene	Number of AA in			LCDR3 AA sequence	Nt mutations	AA mutations
						CDR1	CDR2	CDR3						CDR1	CDR2	CDR3			
1A8	IgG1, κ	23	4-4*07	4*02	3-10*01	10	7	13	CAREFYGGRGVVDSW	34	25	nd							
2L8	IgG1, λ	25	1-46*01	4*02	6-13*01	8	8	14	CARGGISWYVTGFDYW	12	8	L2-14*01	L3*02	9	3	10	CSSYTRSSTWVF	10	8
3I23	IgG1, κ	25	3-23*01	4*02	5-24*01	8	8	16	CAKGVGSDFPTSRILDSW	24	16	K3-20*01	K3*01	7	3	8	CHQYGTSFTF	14	9
4E7	IgG1, κ	15	4-4*02	1*01	6-25*01	9	7	12	CAIGGSASVPTKYW	14	10	K1-39*01	K2*01	6	3	9	CQQSYSSPYTF	4	4
4I23	IgG1, κ	23	3-23*01	4*02	4-17*01	8	8	14	CAKNAGDYAPSPADYW	8	6	K6-21*01	K1*01	6	3	9	CHQSSTLPGTF	4	4
2J3	IgA1, λ	342	4-38-2*02	6*03	2-2*01	9	7	18	CARDRSVVVPAAPLYYMDVW	12	7	L2-23*02	L2*01	9	3	10	CCSYATSTNLLF	11	9
3I3	IgA1, λ	387	4-39*01	5*02	3-3*01	10	7	13	CARHPSWDRSWFDPW	24	17	L2-8*01	L2*01	9	3	10	CSSYVGNNNFAF	13	11
4B19	IgA1, κ	177	3-30-3*01	4*02	4-11*01	8	8	10	CARDLSASFDYW	25	18	K2-30*01	K5*01	11	3	10	CMQGTHWPMFTF	9	6
4C10	IgA1, κ	459	3-20*01	5*01	5-12*01	8	8	17	CARDNRGQRGSSFGWFDSW	24	17	K1-39*01	K1*01	6	3	9	CQQDYITPRTF	12	8
5I2	IgA1, κ	120	1-69*01	4*02	5-12*01	8	8	17	CASNRANRADDYDYYFDYW	21	17	K4-1*01	K1*01	12	3	9	CQQYYSIPRTF	8	7
