# Supplementary material for: Frequent Use of the IgA Isotype in Human B Cells Encoding Potent Norovirus-Specific Monoclonal Antibodies That Block HBGA Binding
Source: PLoS Pathog. 2016 Jun 29;12(6):e1005719. doi: 10.1371/journal.ppat.1005719 (PMC4927092; doi:10.1371/journal.ppat.1005719)
Supplement: S3 Table — Hemagglutination inhibition assays were performed as described previously [14]. In brief, human type O erythrocytes were collected from a healthy adult in Alsever’s buffer, washed twice in Dulbecco’s phosphate-buffered saline (PBS) without Ca2+ or Mg2+, and pelleted by centrifugation at 500 x g for 10 min at 4°C. Monoclonal antibodies (mAb; starting concentration 60 μg/mL) were diluted initially 1:10 in PBS with 0.85% saline (pH 5.5), and then serially 2-fold diluted. Four hemagglutination units (~2 ng) of Norwalk virus VLPs were mixed with the diluted monoclonal antibodies and incubated at room temperature for 30 min. The VLP-mAb mixture was added to an equal volume of 0.5% washed type O erythrocytes in 0.85% saline (pH 6.2) and incubated for 2 h at 4°C. The HAI titer was determined by identifying the reciprocal of the highest dilution of mAb that inhibited hemagglutination by the VLPs. (PDF) [file ppat.1005719.s008.pdf]

Table S3. Reciprocal hemagglutination inhibition titer for isotype swtich variant antibodies (starting concentration 60 µg/mL)

Isotype	3I23	4I23	2J3	4C10	5I2
IgG	320	20	80	640	640
mIgA	2,560	40	80	640	160
dIgA	640	40	160	1,280	640

Hemagglutination inhibition assays were performed as described previously [14]. In brief, human type O erythrocytes were collected from a healthy adult in Alsever's buffer, washed twice in Dulbecco's phosphate-buffered saline (PBS) without Ca^{2+} or Mg^{2+}, and pelleted by centrifugation at 500 x *g* for 10 min at 4 °C. Monoclonal antibodies (mAb; starting concentration 60 µg/mL) were diluted initially 1:10 in PBS with 0.85% saline (pH 5.5), and then serially 2-fold diluted. Four hemagglutination units (~2 ng) of Norwalk virus VLPs were mixed with the diluted monoclonal antibodies and incubated at room temperature for 30 min. The VLP-mAb mixture was added to an equal volume of 0.5% washed type O erythrocytes in 0.85% saline (pH 6.2) and incubated for 2 h at 4 °C. The HAI titer was determined by identifying the reciprocal of the highest dilution of mAb that inhibited hemagglutination by the VLPs.
